# COVID-19 Pandemic and Its Impact on Perinatal Outcomes between Symptomatic and Asymptomatic Women

**DOI:** 10.1155/2022/1756266

**Published:** 2022-07-14

**Authors:** Inas Babic, Faten Alsomali, Sana Aljuhani, Sahar Baeissa, Inam Alhabib, Ebtisam AlAhmari, Magdy Omer, Khalid Alkhalifa

**Affiliations:** ^1^Department of Obstetrics & Gynecology, Prince Sultan Military Medical City, Riyadh, Saudi Arabia; ^2^Medical College, Alfaisal University, Riyadh, Saudi Arabia

## Abstract

Coronavirus disease 2019 (COVID-19) has been increasing among pregnant women worldwide. Its impact on maternal, fetal, and neonatal health is still scarce in the published literature. As a routine COVID-19 prenatal screening has been established for all women requiring hospitalization, it is not clear whether symptomatic women carry worse pregnancy outcomes than those without symptoms. We aimed to analyze perinatal outcomes between symptomatic and asymptomatic women admitted to our center. *Materials and Methods*. A single-center retrospective cohort study was conducted for fourteen months. All pregnant women with positive reverse transcriptase-polymerase chain reaction (RT-PCR) test results for severe acute respiratory syndrome coronavirus 2 (SARS-CoV-2) were enrolled, and their perinatal outcomes were analyzed in two groups based on whether they were symptomatic or not. The primary outcomes were composite adverse fetal, neonatal, and maternal outcomes and their comparison between study groups. *Results*. Out of 209 included COVID-19 positive pregnant women, 62 (30%) presented with one or more infection-related symptoms. Symptomatic women were older, multiparous, carried ≥1 comorbid condition, and attained infection at earlier gestational age (44% vs. 28%; 82% vs. 69%; 28% vs. 16%; and 34 vs. 36 weeks, respectively) (*p* < 0.05), when compared to asymptomatic women, respectively. Maternal composite adverse outcomes were higher in the symptomatic group and showed either one or more outcomes, positive chest radiological findings, requiring hospitalization with oxygen supplementation, or maternal death (8% vs. 0.7%) (*p* < 0.05). Composite fetal and neonatal adverse outcomes such as miscarriage, fetal or neonatal death, admission to neonatal intensive care unit, and neonatal COVID-19 infection were not statistically significant (*p* > 0.05) between symptomatic and asymptomatic women. *Conclusion*. COVID-19 infection among symptomatic pregnant women may carry a higher risk for adverse maternal outcomes. It may be associated with their advanced age and comorbid conditions. Maternal infection-associated symptoms per se likely do not pose an increased risk for adverse fetal or neonatal outcomes.

## 1. Introduction

The worldwide pandemic caused by novel severe acute respiratory syndrome coronavirus 2 (SARS-CoV-2) emerged in December 2019, spread across the world, and became a major international health concern [1]. Based on the research conducted on previous coronavirus outbreaks on humans, such as SARS-CoV and the Middle East respiratory syndrome coronavirus (MERS‐CoV), pregnant women and their unborn babies are specifically vulnerable to poor outcomes [2]. While COVID-19 is a novel coronavirus strain, MERS and SARS have been linked to adverse perinatal outcomes including miscarriage, admission to the intensive care unit (ICU), the need for mechanical ventilation, and the development of disseminated intravascular coagulation (DIC) and ending in maternal death [3, 4].

Currently, SARS-CoV-2 has been inhabiting our lives worldwide. It subsides in one part of the world and returns in a surge in another, each time bringing unknown consequences. Although more data are emerging in the literature every day, still little is known about the relationship between COVID-19 and its effects on mothers and their babies with conflicting findings [5, 6].

Due to physiological transformations that women undergo during gestation, it makes them more susceptible to severe infections. During pregnancy, women's body physiologically tends to undergo an adaptive transformation, particularly in the cardiopulmonary system. The changes often result in enhanced hemodilution, heart rate, stroke volume, and reduced pulmonary residual capacity. The changes are associated with an increased risk of hypoxemia and hypoxia [7]. Concurrently, the general state of low immunity may emerge in the context of host-graft rejection that occurs in the mother-fetus relationship. [7].

However, in general, despite being considered a high-risk population, pregnant women and their neonates often overcome COVID-19 infection with an overall low incidence of adverse outcomes [6, 8, 9].

COVID-19 infection is frequently acquired during gestation. However, women are often asymptomatic, sometimes not aware of the infection and its possible consequences on their health and pregnancy outcomes. In this study, we researched COVID-19 infection and its clinical impact on perinatal outcomes between asymptomatic and clinically symptomatic pregnant women.

As the pandemic is in the surge, numerous health centers worldwide are adapting hospital policies and regulations for routine COVID-19 screening. Our hospital is one among those. An increasing number of asymptomatic, apparently well-pregnant women are being encountered by the health staff revealing positive screening COVID-19 test results. It often provokes anxiety and unpredictable clinical pathways for both patients and the responsible medical team. Our aim was to research whether there is any difference in associated maternal characteristics and their perinatal outcomes between asymptomatic and symptomatic COVID-19 pregnant women.

## 2. Materials and Methods

This is a retrospective cohort study of COVID-19 pregnant women at a single large tertiary center, Prince Sultan Military Medical City in Riyadh, Kingdom of Saudi Arabia. Universal COVID-19 screening was established for all women requiring admission to the obstetric unit in May 2020. From March 2020 to May 2020, only women with exposure history or reporting symptoms (fever, cough, or shortness of breath) were tested for COVID-19. Testing was performed with a nasopharyngeal swab using reverse transcriptase-polymerase chain reaction (RT-PCR) for severe acute respiratory syndrome coronavirus 2 (SARS-CoV-2).

All obstetric admission records were reviewed between March 2020 and May 2021 (14 months) by using an electronic hospital system.

Women with documented PCR COVID-19 positive tests were included in the study and further divided into two groups: symptomatic and asymptomatic, depending on whether they described any COVID-19-associated symptoms or not.

This study was approved by the Central Research Ethics Committee under project no. 2020-021.

We retrieved relevant maternal demographics and pregnancy information including maternal age, parity, BMI, history of medical diseases, time and duration of infection, laboratory tests, radiological tests (chest X-ray, CT chest, and obstetric ultrasound), gestational age, mode, and reason for delivery. COVID-19 symptoms were recorded for all symptomatic women. Additionally, neonatal outcomes include birth weight, APGAR score, admission to neonatal intensive care unit (NICU), duration of hospitalization, and neonatal COVID-19 swab results.

The main outcomes were maternal, fetal, and neonatal adverse outcomes as individual findings and composite outcomes.

Maternal adverse outcomes were a panel of adverse events including severe respiratory symptoms that required hospitalization, ICU admission with oxygen supplementation, and maternal death. Fetal or neonatal adverse outcomes included miscarriage, fetal death (FD), neonatal death (NND), admission to NICU, and confirmed COVID-19 neonatal infection. To be analyzed as composite adverse outcomes, the woman or the fetus/neonate should demonstrate one or more adverse events. Pregnant women, who gave birth outside our center, were not included in the study.

### 2.1. Statistical Analysis

Data were analyzed by using descriptive and inferential statistics via IBM SPSS ® version 20. Continuous variables were checked for normality distribution and expressed as mean (minimum–maximum) for nonparametric variables. Categorical variables were expressed as frequencies (*n*) and percentages of occurrence (%). Mann–Whitney was utilized for nonparametric continuous variables, while McNemar's chi-squared test was used for categorical variables. The significance level was 0.05 for all statistical tests.

## 3. Results

During the period of 14 months, 240 pregnant women tested positive for RT-PCR COVID-19. 31 cases were excluded due to either incomplete data, or the patient was discharged to give birth in another hospital. 147 (70%) were asymptomatic and discovered to have positive screening tests just prior to or upon admission to the hospital.

Symptomatic pregnant women suffered from a wide range of symptoms but generally demonstrated mild clinical presentations. The most frequently described symptoms were cough (64%), fever (38%), and dyspnea (34%). More than two-thirds (71%) reported more than one symptom as shown in Table 1.

Overall, it was shown that COVID-19 infection was more common among multiparous, obese women at an advanced age, above 35. This difference was further prominent when compared between symptomatic and asymptomatic women with statistically significant results (*p* < 0.05) as shown in Table 2. Although more than two-thirds were either obese or overweight, there was no significant difference between the study groups.

Chronic diseases were more commonly encountered among symptomatic women, 28% vs. 16% (*p* < 0.05), with diabetes mellitus leading the incidence of 12% and 4% among symptomatic versus asymptomatic pregnant women, respectively.

Above 90% of women in both groups got infected during the third trimester, following delivery at early term.

Although symptomatic women were diagnosed with infection earlier in pregnancy, with two weeks difference, and most of them stayed longer pregnant and gave birth at early term, with no significant difference when compared with the asymptomatic group.

The rate of preterm birth (20% and 15%) and emergency cesarean sections (64% and 63%) between asymptomatic and symptomatic women did not reach a statistically significant difference (*p* > 0.05).

Obstetric ultrasound for fetal anatomy growth and well-being was performed for all women. There were 8 scans, 4 in each study group, that revealed additional findings. In a symptomatic group, a scan revealed two subchorionic hematomas, open cervix, idiopathic polyhydramnios, and placenta accreta. In the asymptomatic group, obstetric ultrasound revealed one fetus to be small for gestational age, one case of idiopathic oligohydramnios, one fetus was large for gestational age, and one case of kidney abnormalities.

Symptomatic pregnant women suffered from a wide range of symptoms but generally demonstrated mild clinical presentations. The most frequently described symptoms were cough (64%), fever (38%), and dyspnea (34%). More than two-thirds (71%) reported more than one symptom as shown in Table 1.

Maternal outcomes showed a higher incidence of symptomatic women requiring hospitalization and oxygen supplementation (13% versus 2% (*p* < 0.05)). Positive findings on chest X-ray and/or CT commonly revealed patchy shadowing ground-glass opacity among both studied groups but slightly higher in the symptomatic group (23% versus 6%) (*p* < 0.05).

During the study period, in this cohort, there were neither COVID-19 infected women that required intensive care monitoring with ventilation respiratory support nor COVID-19 which directly caused maternal deaths (Table 3.).

Our analysis showed no significant difference in fetal and neonatal outcomes among all infected women, whether they were symptomatic or not, as demonstrated in Table 3. There was a low incidence of miscarriage and fetal death (9% and 2.8%), respectively, with no significant difference between symptomatic and asymptomatic women (*p* > 0.05). There were five cases of fetal death between 26- and 32- week's gestation in both study groups with no significant difference (*p* > 0.05). All fetuses showed unremarkable second-trimester anatomy. No postnatal autopsy or genetics testing was done. There was only one case of trisomy 18, confirmed through invasive prenatal testing that ended with fetal death at 38 weeks of gestation.

The rate of admission to NICU was similar between the groups and mainly required due to prematurity for support and close observation. Our study included two sets of twins that were delivered prematurely and were admitted to the NICU. Both mothers were asymptomatic, and the babies were not COVID-19-infected. There were a total of 10 (5%) neonates diagnosed with COVID-19 following the delivery with no difference between the study groups.

## 4. Discussion

In Saudi Arabia, during 14 months of the COVID-19 pandemic, there were three infection surges, each led by new SARS-CoV-19 mutants. General public health measures with obligatory wearing masks, maintaining social distancing and avoiding gatherings, and including lockdowns have helped with decreasing infection rates over the pandemic course.

In our center with high patient turnover and a large number of deliveries (∼10,000/annum), the incidence of COVID-19 infection among pregnant women in our center remained relatively low, around 2%. SARS-CoV-2 vaccines were available for pregnant women 2 months before ending this study, and none among enrolled cases had been vaccinated. It did not interfere with the results and overall study findings.

Uniformly to some other studies, COVID-19 infection among our study population has been mild, without major consequences on maternal and neonatal lives [10, 11].

Although symptomatic women required additional medical care with a higher incidence of hospitalization, it did not leave a worrisome impact on perinatal outcomes. Their symptoms were mild and often self-limited [12].

As commonly reported elsewhere, high BMI is an associated factor for the development of COVID-19 infection during pregnancy. Women in this cohort were >70% either overweight or obese with no significant difference of whether they were symptomatic or not. These women commonly suffer from chronic vascular diseases, such as diabetes and hypertension, which may act as dominant associated factors for attaining viral infection [13, 14].

Advanced maternal age, >35 and >40, have been identified as an independent risk factor for COVID-19 infection among pregnant women with worse outcomes [15]. Likewise, to a recent large review, we have found that elderly women, either at >35 or >40 years of age were more commonly symptomatic, multiparous, and suffered from one or more comorbidities which ultimately led to their worse maternal outcomes [16].

Expectedly, as demonstrated in some other studies, women with diabetes mellitus, hypertension, and respiratory disease carried a higher risk of symptomatic disease requiring longer hospitalization and longer recovery [13, 17].

Similar to other study populations, women in this cohort commonly got infected during the third trimester, many symptomatic women remained pregnant and continued to carry pregnancy to term but rarely reached the due date.

Similar to some other reviews [18, 19], the incidence of preterm birth was slightly higher among COVID-19 women (19%) but with no significant difference between symptomatic and asymptomatic groups. A small portion of them is iatrogenic, often induced near term due to increased concerns about maternal or fetal health.

Interestingly, a recently published national quasiexperimental study showed a decreased incidence of preterm birth, likely due to several factors, such as decreased physical activity, less iatrogenic premature interventions, and may be less environmental pollutant exposure due to prolonged lockdowns [20, 21].

The rate of cesarean section was higher in the symptomatic group, albeit not reaching a significant difference. We can speculate that COVID-19 infection carries unknown consequences and often does increase both patient and physician's anxiety favoring swift delivery. It frequently leads to a quick decision to perform cesarean section rather than inducing labor which requires a longer period of delivery.

Contrary to many other studies reporting increased maternal adverse outcomes including a high maternal death rate, especially in India and Brazil [22, 23], we had no reported maternal ICU admission requiring intensive monitoring with oxygen supplementation or maternal death. This could be related to the promotion and strict implementation of public health measures, facilities available for treatment, and women's access to the hospitals. There were 8 and 3 women from symptomatic and asymptomatic groups that required isolation in the hospital with oxygen supplementation via mask. All of them recovered within 30 days without consequences to their short-term health.

The rate of neonatal COVID-19 infection was 4.7% in our center with no significant difference of whether the mother is symptomatic or not. All neonates of COVID-19 mothers are separated from their mothers and isolated. Once the neonate is confirmed PCR SARS-CoV-2 test positive, the neonate will be kept separated under close monitoring under the care of the neonatology team.

As maternal samples—vaginal swabs, cord blood, and breast milk—are not routinely tested for COVID-19, the source and route of neonatal infection are not conclusive. We may speculate that vertical transmission is a possible route, but its mechanism is still uncertain.

Although symptomatic mothers were sick for a longer duration and stayed longer pregnant, their neonatal outcomes were rather incredibly reassuring. All babies were discharged home within two weeks of life with no COVID-19 infection-related complications. Similarly, Martinez-Perez et al. have found a low incidence (4.2%) of neonatal COVID-19 infection with resolving symptoms within 10 days [24]. Case series from Wuhan demonstrated favorable neonatal outcomes with no neonatal deaths caused by COVID-19 infection [25]. Furthermore, Vouga et al. reported 2.9% of neonates that tested positive for SARS-CoV-2 with limited knowledge about the route and long-term outcomes [16].

Our study is not without limitations. It is a single-center study setting, retrospective in nature, and small numbers, but as this is a novel viral infection, a robust overview of the disease carries a valuable insight into the disease among our patient population. This study enlightens some features of the disease which are valuable for patients' counseling. Further studies are required to elucidate whether preventative measures, particularly the generalized immunization process among pregnant women, might decrease and eliminate infection for those at risk. Women at risk should be counseled about their potential risks of acquiring the disease and educated on various pathways how to minimize adverse events.

## 5. Conclusion

In this study, we found that symptomatic multiparous women at an advanced age with associated comorbidities are at a higher risk for hospitalization and oxygen support. Infection prevention with decreased exposure risk is crucial whenever possible. Fetal and neonatal outcomes are not significantly worse among women presenting with COVID-19 symptoms in comparison with those who are asymptomatic.

## Figures and Tables

**Table 1:** Characteristics of COVID-19 symptoms among infected pregnant women.

Symptoms	All (209) (%)	Symptomatic (62) (%)
Fever	23 (11)	38
Cough	39 (19)	64
Dyspnea	21 (10)	34
Headache	14 (8.8)	23
Sore throat	14 (8)	23
Diarrhea	6 (3)	10
Anosmia and/or ageusia	2 (1)	3
Nausea and/or vomiting	16 (8)	26
Dizziness	1 (0.5)	2
Rhinorrhea	9 (5)	15
Myalgia	1 (0.5)	1
>1 symptom	44 (21)	71

**Table 2:** Maternal characteristics, investigations, and pregnancy outcomes.

	All 209 (100%)	Asymptomatic 147 (70%)	Symptomatic 62 (30%)	*p* value
Age	31 ± 6.1	30 ± 5.9 (19–47)	32 ± 6.4 (20–45)	0.044
Age >35	68 (33)	41 (28)	27 (44)	0.035
Age >40	20 (10)	10 (7)	10 (16)	0.043
Para 0	56 (27)	45 (31)	11 (18)	0.019
Para 1	47 (23)	31 (21)	16 (26)	
Para >1	104 (50)	69 (48)	35 (56)	
BMI	32.5 ± 6.57	32.44 ± 6.78	32.64 ± 6.06	0.865
Overweight^*∗*^	53 (33)	39 (33)	14 (33)	0.973
Obese^*∗∗*^	112 (68.3)	82 (68.3)	30 (68)	0.985
Hypertension	4 (2)	0	4 (7)	0.002
Diabetes	13 (6)	6 (4)	7 (12)	0.045
Bronchial Asthma	6 (3)	2 (1)	4 (7)	0.041
Composite medical disease^*∗∗∗*^	40 (19)	23 (16)	17 (28)	0.036

*Trimester at diagnosis*				
1st	3 (1)	2 (1)	1 (2)	0.112
2nd	15 (7)	7 (5)	8 (13)	
3rd	191 (91)	138 (94)	53 (86)	
Gestation at diagnosis (weeks ± days)	36 ± 5	36 ± 5	34 ± 5	0.028
Duration of symptoms (days)	1 ± 3 (0–30)	—	5 ± 4.4 (0–30)	—

*Labs*				
WBC	10 (3.1–23.3)	10 (4.6–23.3)	8.7 (3.1–16.0)	0.067
HB	11 (6.5–14.6)	10.93 (6.5–13.9)	11.1 (7.5–14.6)	0.788
PLT	268 (126–582)	267 (126–492)	268 (152–582)	0.690
AST	17 (5–151)	17 (5–151)	17 (5–66)	0.296
ALT	13 (.7–111)	18.9 (1.92–111)	6.5 (0.67–12)	0.590

*Perinatal outcomes*				
Gestation at delivery (weeks) (min-max)	37 ± 3 (11–42)	37 ± 2 (11–42)	37 ± 3 (16-41)	0.415
Duration diagnosis-delivery (days)	12 (0–233)	7 (0–219)	22 (0–233)	0.001
Preterm birth^$^	39 (19)	30 (20)	9 (15)	0.075
Vaginal delivery^$^	120 (58)	88 (61)	32 (54)	0.366 0.380
CS (all)	83 (40)	56 (39)	27 (46)	
Elective CS	30 (36)	20 (36)	10 (37)	
Emergency CS (all)	53 (64)	36 (64)	17 (63)	
NRFH	21 (40)	16 (44)	5 (29)	
FTP	9 (17)	4 (11)	5 (29)	
Other^^^	23 (43)	16 (18)	7 (41)	
Male gender	95 (47)	66 (46)	29 (49)	0.698
Birth weight (g)	2973 ± 575	2980 ± 580	2955 ± 567	0.831
APGAR at 1 min	8 (1–9)	8 (2–9)	8 (1–9)	0.114
APGAR at 5 min	9 (1–10)	9 (3–10)	9 (1–9)	0.097
Positive findings on Ob ultrasound^^^^	8 (5)	4 (3)	4 (8)	0.208

^*∗*^Overweight defined as BMI >25, ^*∗∗*^obese defined as BMI> 30, and ^*∗∗∗*^composite medical disease defined as one or more medical diseases such as hypertension, diabetes, bronchial asthma, systemic lupus erythematosus, and thyroid disease. ^$^Excluded deliveries <24 weeks gestation. ^^^Other reasons for an emergency cesarean section such as abruptio placentae, preeclampsia, or labor with a malpresentation. ^^^^Abnormal fetal growth or amniotic fluid, cervical shortening, or placental abnormalities. *t*-test was used for the analysis of independent variables. Chi-Square and Fisher exact test were used for categorical variables and expressed as frequencies + percentages (%). Percentages are calculated using rounded counts.

**Table 3:** Adverse perinatal outcomes.

Outcomes	All	Asymptomatic	Symptomatic	*p* value	Rr (odds ratio 95% CI)
*Maternal outcomes*					
Hospitalization	11 (5.3)	3 (2.1)	8 (13.1)	0.001	1.12 (1.02–1.24)
Chest XRAY/CT scan (+ve findings)	22 (10.5)	8 (5.5)	14 (22.5)	<0.001	1.22 (1.06–1.40)
ICU admission		0	0		—
Maternal death		0	0		—
Composite maternal adverse outcomes	11 (5.3)	3 (2.1)	8 (13.1)	<0.001	1.12 (1.02–1.24)

*Fetal/Neonatal outcomes*					
Miscarriage	2 (.9)	1 (0.6)	1 (1.6)		—
IUFD	6 (2.8)	4 (2.7)	2 (3.2)	0.842	1.19 (.213–6.68)
Admission to NICU	17 (8.2)	10 (6.8)	7 (11.4)	0.232	—
NICU stay (days) (min-max)	4 (0–40)	4 (2–40)	4 (0–33)	0.368	—
Neonatal Cov19 infection	10 (4.7)	8 (5.9)	2 (3.5)	0.491	0.577 (.119–2.807)
Composite fetal/neonatal adverse outcomes	28 (13.4)	18 (12.2)	10 (16.1)	0.451	1.37 (0.597–3.184)

## Data Availability

All data have been included in the manuscript.
